# P-252. A New Model of HIV Disease Progression Based on the Accumulation of Non-AIDS Comorbidities

**DOI:** 10.1093/ofid/ofaf695.473

**Published:** 2026-01-11

**Authors:** Senay Topal, Vincent Marconi, Joseph Lipscomb, Julia W Gallini, Nusrat J Epsi, Kristopher Kuhn, Xiuping Chu, Amy Justice, Brian Agan

**Affiliations:** Uniformed Services University of the Health Sciences, North Bethesda, MD; Emory University, Atlanta, Georgia; Rollins School of Public Health at Emory University, Atlanta, Georgia; Boston University, Boston, Massachusetts; IDCRP HJF, Bethesda, Maryland; Uniformed Services University, Chesapeake, Virginia; Infectious Disease Clinical Research Program, The Henry M. Jackson Foundation for the Advancement of Military Medicine, Bethesda, Maryland; Yale School of Medicine, West Haven, CT; Infectious Disease Clinical Research Program, Department of Preventive Medicine and Biostatistics, Uniformed Services University of the Health Sciences, Bethesda, MD, USA, Bethesda, Maryland

## Abstract

**Background:**

Although AIDS has declined and life expectancy has improved, people with HIV (PWH) develop more non-AIDS comorbidities than people without HIV (PWoH). Health-related quality of life for PWH is now primarily influenced by non-AIDS comorbidities rather than by AIDS-defining conditions. We propose a model of HIV disease progression that accounts for the challenges faced by PWH today by extending previous models via the inclusion of non-AIDS comorbidities that accumulate over time.

**Figure 1.**
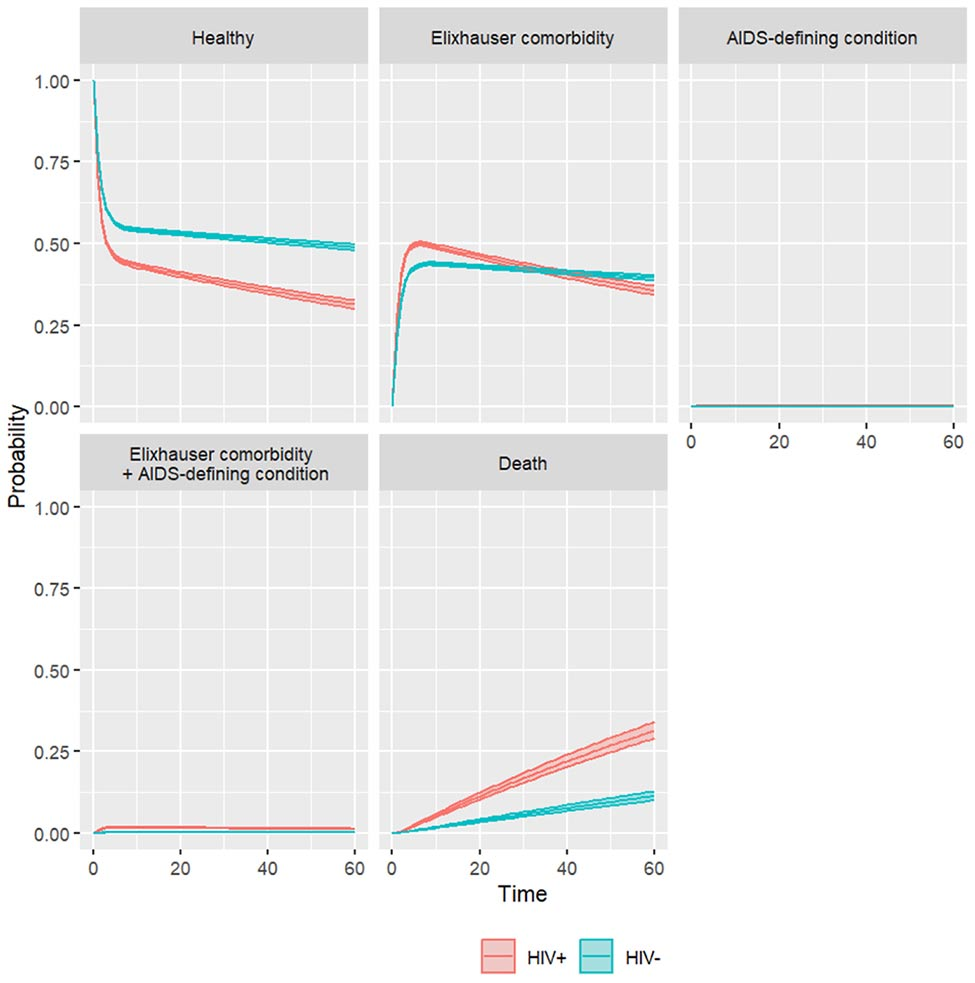
AIDS and non-AIDS conditions among people with and without HIV. Markov state transition model. Time in years.

**Figure 2.**
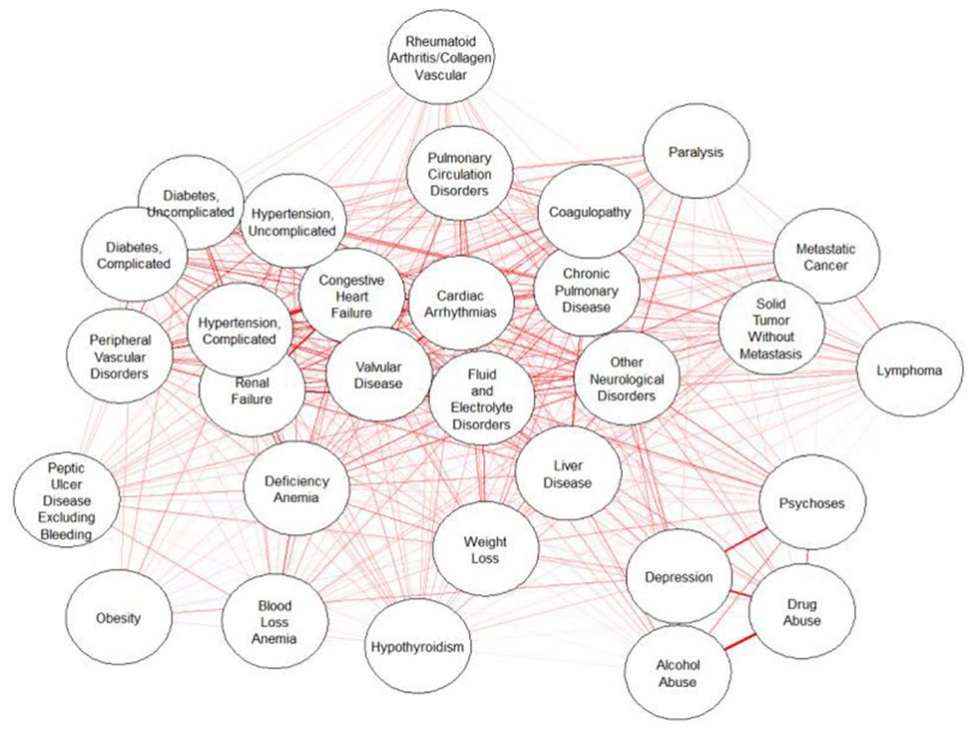
Elixhauser morbidity among people with HIV. Force-directed placement. Line width proportional to pairwise correlation. Overall grouping based on pairwise correlations.

**Methods:**

The sample included 22,945 demographically matched Military Health System beneficiaries with and without HIV of age 18-64 for the post highly active antiretroviral treatment introduction period 2003-2018. This system provides universal access to care, minimizing system-level confounding and isolating patient-level effects. Health states were defined in terms of stages of HIV and non-AIDS Elixhauser comorbidities. Health state preferences were derived using short form 36 data. Markov state transition models were simulated to estimate the probabilities of being in each health state as people age.

**Figure 3.**
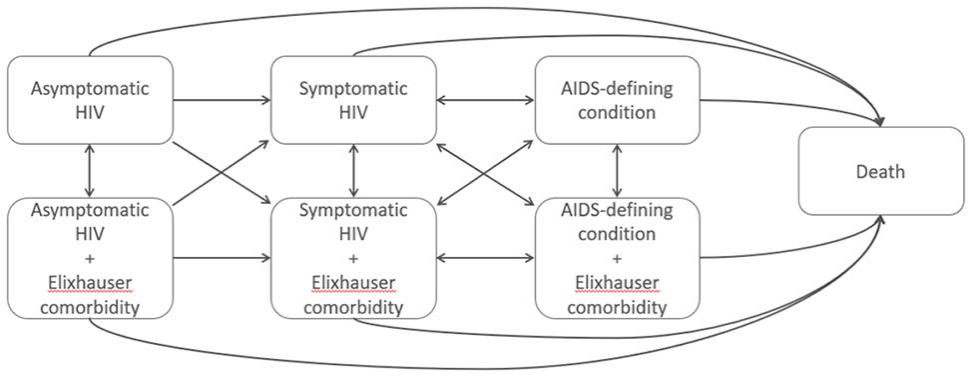
Model of HIV progression with Elixhauser comorbidities.

**Figure 4.**
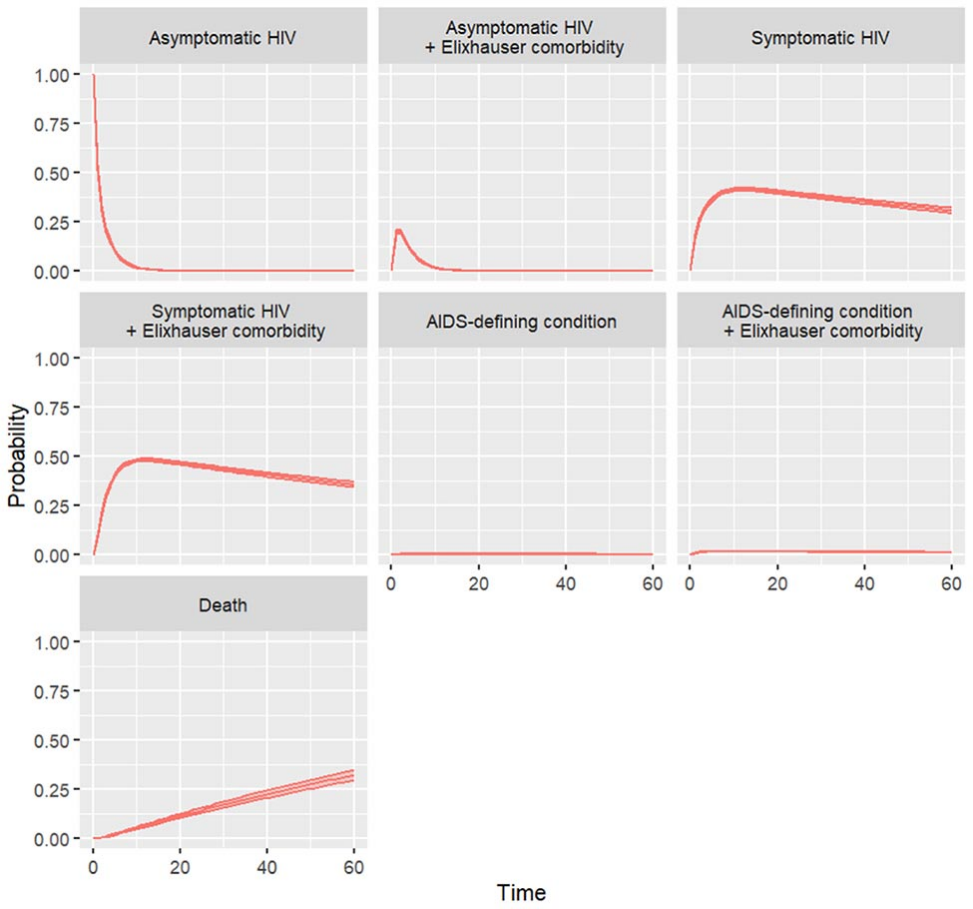
Probabilities of health states of HIV progression with Elixhauser comorbidities. Markov state transition model. Time in years.

**Results:**

Although PWH had higher morbidity than PWoH, this was predominantly driven by non-AIDS comorbidities rather than by AIDS-defining conditions. We found that traditional models of HIV progression, which do not account for non-AIDS comorbidities, can overestimate the preferences of PWH for the states of asymptomatic HIV by up to 24% and of symptomatic HIV by up to 17%, while underestimating that of AIDS by up to 15%. Despite improved HIV treatment, life expectancy of PWH continued to lag behind that of PWoH by 7 years. Given the time spent in each state, traditional models can overestimate quality of life for PWH by up to 6 quality-adjusted life-years.

**Conclusion:**

Traditional models of HIV progression erroneously emphasize AIDS, overlooking the growing burden of non-AIDS comorbidities on health-related quality of life. Blinder–Oaxaca decomposition analyses indicated the higher mortality among PWH, despite the rarity of AIDS, may be largely attributable to this misplaced focus. Non-AIDS comorbidities must be accounted for appropriately in clinical and economic valuations of treatment for PWH.

**Disclosures:**

Vincent Marconi, MD, Lilly: Grant/Research Support|Merck: Grant/Research Support

